# Role of MR-proADM in the risk stratification of COVID-19 patients assessed at the triage of the Emergency Department

**DOI:** 10.1186/s13054-021-03834-9

**Published:** 2021-11-26

**Authors:** Marilena Minieri, Vito N. Di Lecce, Maria Stella Lia, Massimo Maurici, Sergio Bernardini, Jacopo M. Legramante

**Affiliations:** 1 Department of Experimental Medicine and Unit of Laboratory Medicine, Tor Vergata University Hospital, University of Rome Tor Vergata, Rome, Italy; 2 Emergency Medicine, Emergency Department, Tor Vergata University Hospital, Rome, Italy; 3 Unit of Laboratory Medicine, Tor Vergata University Hospital, Rome, Italy; 4 Department of Biomedicine and Prevention, University of Rome Tor Vergata, Rome, Italy; 5 Department of Systems Medicine and Emergency Medicine, Emergency Department, Tor Vergata University Hospital, University of Rome Tor Vergata, Rome, Italy

During the severe acute respiratory syndrome coronavirus 2 (SARS-CoV-2) pandemic, the Emergency Departments have been overrun with suspicious COVID-19 patients, creating a pressing need to optimize resources through risk stratification already at the triage level.

Mid-regional proadrenomedullin (MR-proADM), a more stable fragment of the rapidly degrading active adrenomedullin (ADM) peptide, has been proven a promising biomarker effective in predicting severity and long-term adverse outcomes in pneumonia [1].

Interestingly, when the microcirculatory integrity is deteriorated causing the capillary leak, an alteration of the endothelium barrier function can occur, as during sepsis. It has been demonstrated that in these conditions MR-proADM plasma concentrations tend to increase [2]. Accordingly, Hupf et al. have recently reported high adrenomedullin RNA blood expression in patients with severe COVID-19 disease [3].

In this context, Li et al. hypothesized that the integrity of the epithelial–endothelial barrier was severely interrupted in critical patients with COVID-19-related pneumonia, thus introducing the concept of “viral sepsis” [4].

Although recent studies have demonstrated a predictive value of MR-proADM in critically ill patients with COVID-19-related pneumonia [5], no data are currently available about the risk stratification of patients with a suspected SARS-CoV-2 infection at the triage in the Emergency Department (ED).

The aim of this study was to assess the role of MR-proADM in stratifying the in-hospital mortality risk of COVID-19 triaged patients.

Data from 321 consecutive adult patients (aged > 18 years) admitted at the Emergency Department with a confirmed COVID-19 infection were analyzed. The epidemiological, demographic and clinical data were extracted from the electronic clinical records (Table 1). The study was approved by the local ethics committee (approval number 87/20).

**Table 1:** Demographic and clinical parameters

	Overall	Survivors	Non-survivors	*P* value
	*N* = 321	*N* = 224	*N* = 97	
*Age*				
Years, mean (SD)	63.3 (14.7)	59.6 (14.6)	71.9 (11.2)	< 0.001
*Sex*				
Male, *N* (%)	215 (67.0)	145 (64.7)	70 (72.2)	0.193
Female, *N* (%)	106 (33.0)	79 (35.3)	27 (27.8)	
*Comorbidities*				
Hypertension, *N* (%)	131 (40.8)	70 (31.3)	61 (62.9)	< 0.001
Diabetes, *N* (%)	42 (13.1)	19 (8.5)	23 (23.7)	< 0.001
Respiratory disease, *N* (%)	28 (8.7)	14 (6.3)	14 (14.4)	0.017
Malignancy, *N* (%)	19 (5.9)	10 (4.5)	9 (9.3)	0.093
Cardiovascular disease, *N* (%)	55 (17.1)	27 (12.1)	28 (28.9)	< 0.001
Renal disease, *N* (%)	51 (15.9)	13 (5.8)	38 (39.2)	< 0.001
Obesity, *N* (%)	15 (4.7)	8 (3.6)	7 (7.2)	0.155

Values expressed in percentages (%) indicate the proportion of patients within each group for each variable. Data are presented as mean (standard deviation, SD) where specified. The Chi-square (*χ*^2^) test was used to determine significance between the groups for categorical variables, Student’s t test for the variable of age

Blood examinations were done for mid-regional proadrenomedullin (MR-proADM; normality value < 0.5 nmol/L), C-reactive protein (CRP; < 5 mg/L), procalcitonin (PCT; < 0.5 ng/mL), D-dimer (< 500 ng/mL), lactate dehydrogenase (LDH; < 220 IU/L).

The endpoint was the overall in-hospital mortality. Associations between candidate variables and endpoint were assessed using both univariate and multivariate Cox regression analyses. The discriminatory power of the analyzed variables for predicting mortality was tested by means of a receiver operating characteristic (ROC) curve analysis with area under the ROC curve (AUC) determination.

In COVID-19 patients, MR-proADM assessed at the ED admission showed a good discrimination performance for in-hospital mortality (AUC 0.85) with the optimal cutoff of 1105 as obtained with the Youden index. ROC curves and AUC resulted significantly greater for MR-proADM as compared to the other biomarkers for the primary endpoint, i.e., in-hospital mortality, except for CRP (Fig. 1).

**Fig. 1:**
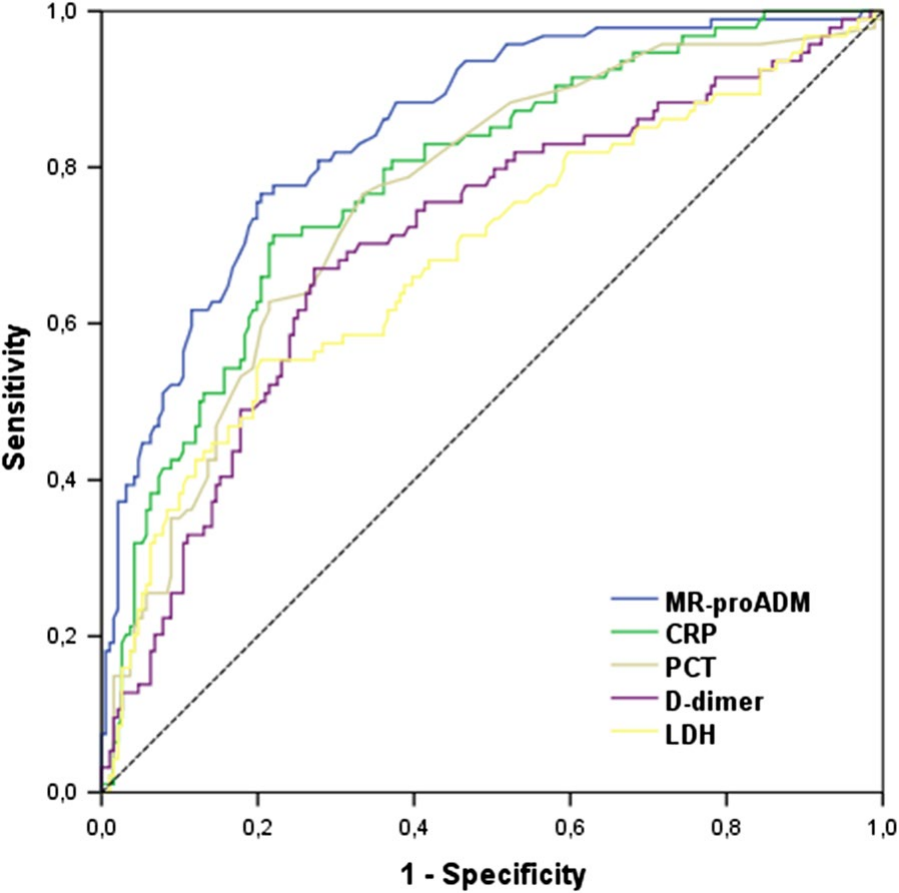
Association of candidate biomarkers with mortality: AUROC area under the receiver operating characteristic curve. MR-proADM, mid-regional proadrenomedullin; CRP, C-reactive protein; PCT, procalcitonin; LDH, lactate dehydrogenase

Pooling together both clinical and laboratory variables in a multivariate analysis and considering the whole observation period, all biomarkers, except PCT, seems to play a key role in the mortality risk stratification at the admission in the Emergency Department. In fact, patients with a value of MR-proADM higher than the cutoff value of 1.105 show a threefold increase in mortality (OR 2.97; IC 1.7–5.28).

To our knowledge, this is the first study focused on the ability of new, as MR-proADM, and traditional biomarkers in the risk stratification of patients with COVID-19 infection at the Emergency Department admission. In particular, our results suggest that above all the MR-proADM, among the other biomarkers analyzed, might play a key predictive role in the early risk stratification of patients with COVID-19 infection. This relevant information might greatly contribute to optimize the hospital resources and to hasten the decision-making process of the emergency physician.

## Data Availability

Yes.
